# Doping Engineering for Optimizing Piezoelectric and Elastic Performance of AlN

**DOI:** 10.3390/ma16051778

**Published:** 2023-02-21

**Authors:** Xi Yu, Lei Zhu, Xin Li, Jia Zhao, Tingjun Wu, Wenjie Yu, Weimin Li

**Affiliations:** 1 School of Microelectronics, Shanghai University, Shanghai 201899, China; 2 State Key Laboratory of Functional Materials for Informatics, Shanghai Institute of Microsystem and Information Technology, Chinese Academy of Sciences, Shanghai 200050, China; 3 Shanghai Institute of IC Materials Co., Ltd., Shanghai 201899, China

**Keywords:** first-principles calculation, high-throughput, aluminum nitride, piezoelectric coefficient, elastic modulus

## Abstract

The piezoelectric and elastic properties are critical for the performance of AlN-based 5G RF filters. The improvement of the piezoelectric response in AlN is often accompanied by lattice softening, which compromises the elastic modulus and sound velocities. Optimizing both the piezoelectric and elastic properties simultaneously is both challenging and practically desirable. In this work, 117 X_0.125_Y_0.125_Al_0.75_N compounds were studied with the high-throughput first-principles calculation. B_0.125_Er_0.125_Al_0.75_N, Mg_0.125_Ti_0.125_Al_0.75_N, and Be_0.125_Ce_0.125_Al_0.75_N were found to have both high *C*_33_ (>249.592 GPa) and high *e*_33_ (>1.869 C/m^2^). The COMSOL Multiphysics simulation showed that most of the quality factor (*Q_r_*) values and the effective coupling coefficient (*K_eff_*^2^) of the resonators made with these three materials were higher than those with Sc_0.25_AlN with the exception of the *K_eff_*^2^ of Be_0.125_Ce_0.125_AlN, which was lower due to the higher permittivity. This result demonstrates that double-element doping of AlN is an effective strategy to enhance the piezoelectric strain constant without softening the lattice. A large *e*_33_ can be achieved with doping elements having d-/f- electrons and large internal atomic coordinate changes of *du/dε*. The doping elements–nitrogen bond with a smaller electronegativity difference (*ΔEd*) leads to a larger elastic constant *C*_33_.

## 1. Introduction

Piezoelectric materials, which can be applied to Radio Frequency (RF) filters, have drawn much attention with the commercialization of 5G communication technologies [1,2,3,4]. Aluminum nitride with wurtzite structure (w-AlN) is the prevailing piezoelectric material for the body acoustic wave (BAW) filters owing to the advantages of high acoustic velocity, minimal acoustic loss, high thermal stability, and good compatibility with Complementary Metal Oxide Semiconductor (CMOS) technology [5,6]. The critical parameters to evaluate the performance of piezoelectric materials for 5G filters are the mechanical quality factor (*Q*) and the longitudinal electromechanical coupling constant (*k*_33_^2^). The higher the *Q*, the lower the mechanical loss. The higher the *k*_33_^2^, the larger the frequency bandwidth. In general, the *Q* value of 5G RF filters based on w-AlN thin film (*Q* = 400) is higher than that based on ZnO thin film (*Q* = 350), achieving low acoustic loss [7]. However, the *k*_33_^2^ (6.1%) [8] of undoped w-AlN is lower than some well-known piezoelectric materials, such as lead zirconate titanate perovskite (PZT) (*k*_33_^2^ = 8–15%) [8] and ZnO (*k*_33_^2^ = 7.5%) [8]; therefore, undoped w-AlN needs further optimization [9].

As shown in Equations (1) and (2), the characteristic *Q* and *k*_33_^2^ of a BAW RF filter are affected by the piezoelectric strain constant (*e*_33_) and elastic constant (*C*_33_) of the piezoelectric material [10,11,12],

(1) $$\frac{1}{k_{33}^{2}}=\frac{C_{33}\varepsilon_{33}^{s}}{e_{33}^{2}}+1,$$

(2) $$Q=\frac{C_{33}+e_{33}^{2}/\varepsilon_{33}^{s}}{\omega \eta_{33}},$$

where *Q*, *ε*_33_*^s^, ω*, and *η*_33_ are the acoustic quality factor, the clamped permittivity, the angular frequency, and the viscosity coefficient (details are shown in the support information) along the c-axis direction, respectively. A high *C*_33_ is favorable to *Q*, and a high *e*_33_ is favorable to *k*_33_^2^. The piezoelectric material coupling coefficient *k*_33_^2^ and resonator effective coupling coefficient *K_eff_*^2^ are positively related. It is not hard to design a resonator with a high *K_eff_*^2^ from a material having a high *k*_33_^2^ value [13].Consequently, w-AlN should be tailored to have a high *C*_33_ and *e*_33_, simultaneously, which has been proven to be a difficult task.

For example, first-principles calculations [14] and experiments [15] showed that an ~400% increase in the piezoelectric coefficient (*d*_33__≈_ *e_33/_C_33_*) of w-AlN can be achieved with Sc doping. The increase in the *e*_33_ is caused by the increase in the sensitivity of the internal atomic coordinates in response to the strain (*du/dε*) [16]. However, there also exists an elastic softening, owing to the elongated energy landscape in the *c/a* direction [17]. The *e*_33_ of w-X_a/2_Y_a/2_Al_1−a_N (X = Li; Y = V, Nb, Ta; a = 0.125, 0.25, 0.375) is enhanced compared to that of undoped w-AlN [18], while the *C*_33_ decreases simultaneously due to the fact that these dopants can lead to a phase transition to a non-polar hexagonal structure. Hirata et al. [19] used first-principles calculations to investigate the enhancement in piezoelectric properties and the reduction in elastic properties by co-doping w-X_a/2_Y_a/2_Al_1−a_N (X = Mg; Y = Nb, Ti, Zr, Hf; a = 0.125). The bonding analysis of the metal–nitrogen pairs by co-doping Mg + Y into w-AlN was carried out by the crystal orbital Hamilton population (COHP), which showed that weaker bonding energy is one of the reasons for the elastic softening.

The above results showed the need for a new mechanism to achieve a high *C*_33_ and *e*_33_ simultaneously. Manna et al. [20] found that the co-doping of Y and B elements in w-AlN improved the elastic properties while retaining good piezoelectric performance. Subsequently, Jing et al. [21] discovered that the *C*_33_ of B_0.125_Sc_x-0.125_Al_1−x_N is higher than that of Sc_x_Al_1−x_N with a small enhancement of the *e*_33_. These results confirm the feasibility of improving the piezoelectric and elastic properties by dual-element co-doping [22,23]. However, there is still a lack of systematic analysis leading to a clear strategy to choose doping elements for the enhancement of both the *C*_33_ and *e*_33_. Therefore, expanding the map of doping elements and the understanding of the adjustment mechanism is critical to finding new doping schemes with excellent performance.

In this work, a high-throughput workflow is designed to calculate the piezoelectricity and elasticity of 117 X_0.125_Y_0.125_Al_0.75_N compounds. Filtered by the non-magnetic criteria, semiconductor criteria, stability criteria, and performance criteria, three dopants are finally screened out, which are B_0.125_Er_0.125_Al_0.75_N (*e*_33_ = 2.11 C/m^2^, *C*_33_ = 262.2 GPa, *d*_33_ = 8.05 pC/N), Mg_0.125_Ti_0.125_Al_0.75_N (*e*_33_ = 2.41 C/m^2^, *C*_33_ = 261.1 GPa, *d*_33_ = 9.22 pC/N), and Be_0.125_Ce_0.125_Al_0.75_N (*e*_33_ = 2.12 C/m^2^, *C*_33_ = 272.0 Gpa, *d*_33_ = 7.78 pC/N). All have higher piezoelectric and elastic properties than Sc_0.25_Al_0.75_N (*e*_33_ = 1.87 C/m^2^, *C*_33_ = 249.59 GPa, *d*_33_ = 7.49 pC/N). It is found that the primary factor influencing the *C*_33_ is the electronegativity difference (*ΔEd*) of the metal–nitrogen bonds, and the primary factor influencing the *e*_33_ is the *du/dε* of the doping atoms. The bonds with a small *ΔEd* in the doped-AlN between the doping elements and nitrogen with stronger strength leads to a larger elastic constant *C*_33_. The energy competition between the doping atoms and Al mainly affects the internal structural response (*du/dε*) of the crystal due to the transition elements doping into tetrahedral Al sites, tending to form non-tetrahedral coordinates, and undergoing excursions. The increasing of *C*_33_ from the electronegativity difference and *e*_33_ from the *du/dε* of the doping atom with d-/f- electrons provides clear ideas to design new piezoelectric materials for 5G filters.

## 2. Computational Details

**Figure 1:**
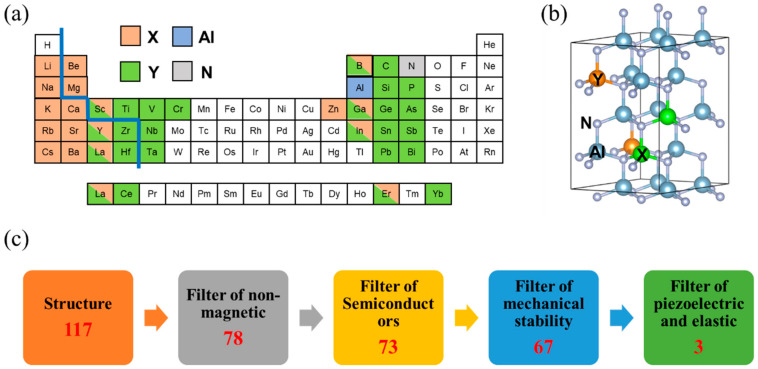
High-throughput workflow of screening piezoelectric material w-X_0.125_Y_0.125_Al_0.75_N. (**a**) Dopants considered in this study. The blue line separates elements X and Y according to an *ΔEd* less than/more than 1.7. (**b**) Crystal structure of w-X_0.125_Y_0.125_Al_0.75_N. (**c**) High-throughput workflow of screening X_0.125_Y_0.125_Al_0.75_N with a high *e*_33_ and high *C*_33_. The red numbers indicate the number of remaining systems after screening.

The 2 × 2 × 2 supercells for w-X_0.125_Y_0.125_Al_0.75_N (Figure 1b) were built with the special quasi-random structures (SQS) method [24]. The first-principles calculations were performed with the Vienna Ab initio Simulation Package (VASP) [25,26,27]. The Perdew–Burke–Ernzerhof (PBE) type generalized gradient approximation (GGA) as the exchange–correlation function was implemented [24]. The elastic tensor was determined by performing the finite differences method. Six finite distortions of the lattice were taken, and the corresponding elastic constants could be derived from the strain–stress relationship [28]. The strains for the original structure along each of the Cartesian directions were ±0.5% and ±1%. The piezoelectric tensors were evaluated from the phonon and dielectric response calculations performed from the density functional perturbation theory (DFPT) [29,30,31]. The Monkhorst−Pack method [32] was used to set the k-point mesh. The k-grids used in the calculation of the structural optimization, self-consistent, and *C_ij_*/*e_ij_* were 30/L+1, 60/L+1, and 30/L+1, respectively, where L is the lattice constant of the systems. The cutoff energy of all calculations was 520 eV. The convergence criteria for the energy and force were set to 10^−4^ eV and 10^−2^ eV/Å, respectively. The Hubbard U values were from Wang et al. and Dudarev et al. [33,34].

The two-dimensional sandwich structure of the resonator and its geometric parameters is shown in Figure S1. The resonator consists of a piezoelectric material with top and bottom electrodes. COMSOL Multiphysics 6.0 is used to simulate the resonator quality factor(*Q_r_*) and effective electromechanical coupling coefficient (*K_eff_*^2^) of the resonator by using the finite element method [35]. Among them, the 2nd order Taylor approximation was performed to simulate the *K_eff_*^2^ [36]. The *Q_r_* value was calculated using the method proposed by Bode et al. [37]. The physical parameters of the materials utilized in the simulation are shown in Table S1.

## 3. Results

To explore the theoretical feasibility of doping engineering to obtain materials with a high performance of large *e*_33_ and *C*_33_, 117 dopants of X_0.125_Y_0.125_Al_0.75_N without toxic elements were tested. As shown in Figure 1a, the orange, green, blue, and gray spheres indicate X, Y, Al, and N, respectively. Considering the charge conservation law, the reasonable elements X and Y are substituted to the Al sites by 1:1. Moreover, Sc, Y, La Er, B, Ga, and In elements can be doped into either the X site or Y site due to the valence of +3. To effectively screen the piezoelectric and elastic performance of X_0.125_Y_0.125_Al_0.75_N materials, a high-throughput workflow was designed (Figure 1c). First, the entries with complex magnetism were removed due to the difficulties to accurately calculate the properties of the magnetic materials for the high-throughput method. Second, the non-semiconductor systems were removed. If the band gap of X_0.125_Y_0.125_Al_0.75_N is less than 0, it indicates that the system is metallic and is not suitable for making piezoelectric layers for 5G filters. Then, the mechanical criterion was tested by the Born–Huang criteria of hexagonal structures [38]: *C*_11_ > *C*_12_, 2*C*_13_^2^ < *C*_33_ (*C*_11_ + *C*_12_), *C*_44_ > 0, *C*_66_ > 0. It is clear that all of the models we considered were mechanically stable, and the detailed results are listed in Table S2. Finally, three dopants (B_0.125_Er_0.125_Al_0.75_N, Mg_0.125_Ti_0.125_Al_0.75_N, and Be_0.125_Ce_0.125_Al_0.75_N) were screened out as having better performance than Sc_0.25_Al_0.75_N. For comparison purposes, the calculation results of Sc_0.25_Al_0.75_N were *e*_33_ = 1.87 C/m^2^, *C*_33_ = 249.59 GPa, and *d*_33_ = 7.49 pC/N, consistent with the results reported by Caro et al., Tasnadi et al., etc. [14,15,39,40,41]. (Details can be found in Figure S2).

The detailed results of the 67 mechanically stable dopants are shown in Table 1. The modulation ranges of the *e*_33_ and *C*_33_ are 0.064~2.408 C/m^2^ and 165.556~396.671 GPa, respectively. Table 1 shows that the *e*_33_ of the dopants with small atomic radii elements and transition elements is high. The *e*_33_ of the dopants with large atomic radii, such as K, Rb, Ca, Sr, Ba, and La, is smaller than that of those with small atomic radii, such as Li and Mg. Furthermore, the dopants that have one small radii element and one transition element (e.g., Mg co-doped with Ce, Ti, Hf, and Zr) show a higher *e*_33_ than Mg co-doping with carbon group elements (i.e., C, Si, Ge, Sn, and Pb). For the *C*_33_, when the difference between the electronegativity of the doping atom and the N element is small, the *C*_33_ is always high. For example, Be_0.125_C_0.125_Al_0.75_N has an *ΔEd* = 0.98 and a *C*_33_ = 346.605 GPa. Comprehensively considering the *e*_33_ and *C*_33_, B_0.125_Er_0.125_Al_0.75_N, Mg_0.125_Ti_0.125_Al_0.75_N, and Be_0.125_Ce_0.125_Al_0.75_N, all having non-transition elements and a small atomic radii atom with a small *ΔEd* and transition elements co-doping, have good performance. It is worth noting that Li_0.125_Ta_0.125_Al_0.75_N, Mg_0.125_Hf_0.125_Al_0.75_N, and Mg_0.125_Zr_0.125_Al_0.75_N, which have a *C*_33_ only somewhat smaller than Sc_0.25_Al_0.75_N and both an *e*_33_ and a *d*_33_ larger than Sc_0.25_Al_0.75_N, are also excellent choices. Better performance can be expected if the doping concentration is further regulated.

## 4. Discussion

### 4.1. Analysis of Elastic Properties

**Figure 2:**
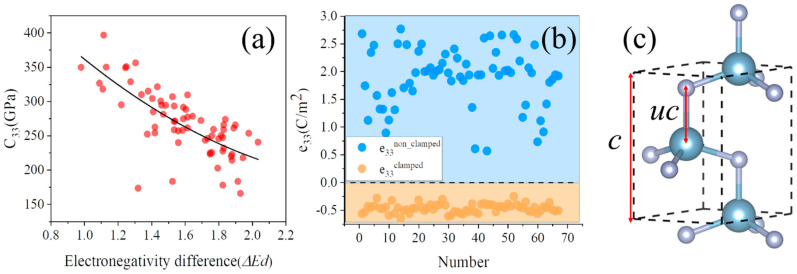
(**a**) The relationship of the *C*_33_ and electronegativity difference. The result of the quadratic fitting is shown as a solid line. (**b**) The *e*_33_*^non_clamped^* and *e*_33_*^clamped^* of X_0.125_Y_0.125_Al_0.75_N. (**c**) Wurtzite structure with the internal parameter *u* = *uc*/*c*.

As shown in Figure 2, we explored in detail the mechanism of co-doping to enhance the characteristics of the *C*_33_ and *e*_33_, respectively. The hardness of the crystal is positively related to the bond density and negatively related to the ionicity indicator *f_i_* [42,43,44]. Figure 2a is the relationship of the *C*_33_ and the electronegativity difference *ΔEd*,

(3) $$\Delta Ed=\frac{\left(E_{X}+E_{Y}-2E_{N}\right)}{2},$$

where *E_X_*, *E_Y_*, and *E_N_* are the electronegativity of elements *X*, *Y*, and *N*, respectively. The electronegativity difference indicates the ionicity indicator (*f_i_*) of the chemical bonds according to the Pauling for AB-type compounds [45],

(4) $$f_{i}\%=(1-e^{-\frac{1}{4}{\left(\Delta Ed\right)}^{2}})\times 100,$$

where *f_i_* indicates the degree of ionization of the hybrid bonds with a larger *f_i_* indicating that the chemical bond is closer to an ionic bond. Figure 2a shows that the *C*_33_ is negatively related to the *ΔEd* (i.e., the smaller the difference of electronegativity, the smaller the *f_i_* and the larger the *C*_33_). Moreover, other factors, such as the bond density induced by lattice distortion, also slightly influence the *C*_33_. A specific mechanistic explanation of the effect of lattice distortion on the *C*_33_ can be found in the supporting information. Generally, the smaller the electronegativity difference, the smaller the degree of ionization of the metal-N in X_0.125_Y_0.125_Al_0.75_N and the larger the hardness of the crystal. Thus, the electronegativity difference could be a criterion for the selected doped-AlN with a high *C*_33_.

### 4.2. Analysis of Piezoelectric Properties

Figure 2b shows the distribution of the *e*_33_, which comprises an electronic-response part and ion-polarization part [46].

(5) $$e_{33}={e_{33}}^{\mathrm{clamped}}+{e_{33}}^{\mathrm{non\_clamped}}$$

*e*_33_*^clamped^* represents the electronic response under strain, which is evaluated by fixing the internal atomic coordinates at their equilibrium positions. *e*_33_*^non_clamped^* represents the ion polarization under strain, which is derived from the internal atomic coordinate changes. The mean and standard deviation of the *e*_33_*^non_clamped^* are 2.001 and 0.689, respectively. However, the mean and standard deviation of the *e*_33_*^clamped^* are −0.435 and 0.077, respectively. Obviously, the *e*_33_*^non_clamped^* mainly contributes the *e*_33_ of w-AlN, owing to wider adjustable values and larger weights. Here, we focus on the derivation of the ion-polarization part,

(6) $${e_{33}}^{\mathrm{non\_clamped}}=\sum_{n}\frac{2eZ_{33}\left(n\right)}{\sqrt{3}a^{2}}\frac{du\left(n\right)}{d\varepsilon },$$

where *n* runs on all atoms in the supercell, *e* is the elementary charge, and *a* is the equilibrium lattice constant. *Z*_33_ is the *c*-axis component of the dynamic Born charge tensor, and *du/dε* is the strain sensitivity. *u* is the ratio of the length of the metal-N along the *c*-axis (*uc*) to the lattice constant *c* in w-AlN (Figure 2c), which can be changed by the strain in the *c* direction. *du/dε* is the factor about the c-structure change, and *Z*_33_ is the factor about the piezoelectric polarization variation on the structure change. Based on the first-principles calculation, the average *Z*_33_ is 2.77 and can be adjusted from −6.48% to 8.53%; the average *du/dε* is 0.17 and can be adjusted from −90.89% to 27.10%. The variation of the *du/dε* is particularly large, which may significantly affect the *e*_33_*^non_clamped^* [16,47].

**Figure 3:**
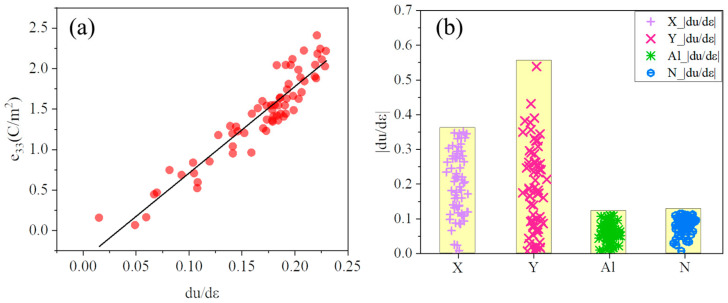
(**a**)The relationship of the *e*_33_ and *du/dε*. *du/dε* is measured by calculating the response of the *u*(*n*) under a macroscopic strain (*η* = 0.5%). The result of the linear fitting is shown as a solid line. (**b**)The |*du/dε*| of X, Y, Al, and N in X_0.125_Y_0.125_Al_0.75_N alloys.

Figure 3a shows that there is a linear correlation between the *du/dε* along the c-axis and the *e*_33_. The *du/dε* of w-AlN is calculated by varying the doping atoms with an adjustment of the internal structure parameter, especially the structure parameter along the *c*-axis. For example, Mg_0.125_Ti_0.125_Al_0.75_N, Li_0.125_Ta_0.125_Al_0.75_N, and B_0.125_Er_0.125_Al_0.75_N have large distortions along the c-axis with a *du/dε* = 0.221, 0.224, and 0.225, respectively, and an *e*_33_ reaching 2.41, 2.24, and 2.11 C/m^2^, respectively. In contrast, Mg_0.125_Ge_0.125_Al_0.75_N, Li_0.125_Sb_0.125_Al_0.75_N, and B_0.125_Ga_0.125_Al_0.75_N have a small distortion along the c-axis with a *du/dε* of 0.178, 0.0152, 0.152, respectively, and an *e*_33_ of only 1.492, 0.155, and 1.202 C/m^2^, respectively. Figure 3b shows that the variation range of |*du/dε*| of the doping elements X and Y is much larger than that of Al and N. The average |*du/dε*| of the doping elements X and Y is 0.195 and 0.184, respectively, while that of the elements Al and N is only 0.0597 and 0.0836, respectively. Thus, the doping elements affect the *e*_33_ dominantly compared to Al and N. The systems with large lattice distortion are doped by Sc, Y, and other transition elements with d-electrons and f-electrons.

**Figure 4:**
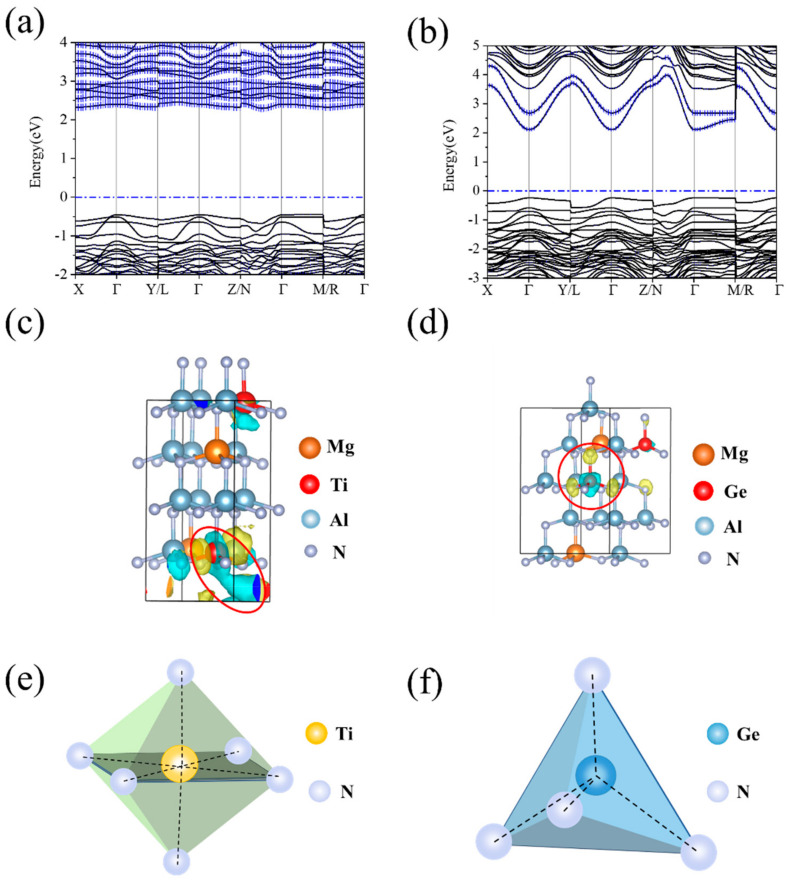
(**a**,**b**) Band structures of Mg_0.125_Ti_0.125_Al_0.75_N and Mg_0.125_Ge_0.125_Al_0.75_N. The blue error bar respectively represents the contribution of the d-electrons of Ti and s-electrons of Ge. (**c**,**d**) Wave function analyses of Mg_0.125_Ti_0.125_Al_0.75_N and Mg_0.125_Ge_0.125_Al_0.75_N are shown in red circles. Blue represents bonding orbitals, and yellow represents anti-bonding orbitals. (**e**,**f**) The structure of Ti_3_N_4_ (Octahedral coordinates) and Ge_3_N_4_ (Tetrahedral coordinates).

To further discuss the mechanism of transition elements affecting the lattice distortion, the band structures and wave functions of the Mg_0.125_Ti_0.125_Al_0.75_N and Mg_0.125_Ge_0.125_Al_0.75_N system were calculated. The doping atoms replace the Al sites, thus the valence band of the undoped and doped w-AlN are all the p-electrons of the N atom. The doping atoms mainly change the electronic state of the conduction band. As shown in Figure 4a,b, the conduction bands of Mg_0.125_Ti_0.125_Al_0.75_N and Mg_0.125_Ge_0.125_Al_0.75_N are occupied by the d-electrons of Ti and s-electrons of Ge, respectively. The sp3 hybridization of w-AlN leads to a tetrahedral coordination geometry of Al; in addition, the doping atoms only have s- and p- electron orbitals (e.g., tetrahedral coordination of Figure 4f). For the transition elements X or Y, such as Ti, Zr, Hf, Er, and Ta, they tend to format other non-tetrahedral coordination (e.g., octahedral coordination of Figure 4e). Octahedral coordination will compete against the tetrahedral coordination of the substituted Al and is more unstable than the tetrahedral coordination of Al. Figure 4c,d shows the wave functions of the conduction band minimum of Mg_0.125_Ti_0.125_Al_0.75_N and Mg_0.125_Ge_0.125_Al_0.75_N. As shown in Figure 4d, for a non-transition element, the electron cloud of the regular tetrahedron geometry to bond to the nitrogen atom does not aggregate in the c-axis. For a transition element, it might bond to the nitrogen atom along the c-axis (Figure 4c). When a strain is performed on Mg_0.125_Ti_0.125_Al_0.75_N with unstable coordination, atoms move away from their regular tetrahedral positions and induce a larger du, which is due to the bond along the c-axis. As a result, the non-tetrahedral coordination of transition elements X or Y is easier to increase |*du/dε*| than the main group doping atoms with tetrahedral coordination under sp3 hybridization. It should be noted that the atomic radius also affects the *e*_33_. While the atomic radius of the doping atom is excessively large, it will produce a large local distortion in the lattice leaving a small space for an atom to move under the strain. For example, in Ba_0.125_Ti_0.125_Al_0.75_N, the atomic radius of Ba is 2.78 Å, and the *du/dε* is only 0.139. In a word, a small atomic radius and d/f-electrons are two parameters for finding doped-AlN with a large *e*_33_.

As shown in Table 2, the *Q_r_* value of all three selected systems is higher than that of Sc_0.25_Al_0.75_N. The trends are the same for the *k*_33_^2^ about co-doped w-AlN material and the *K_eff_*^2^ about the resonator. The *K_eff_*^2^ and *k*_33_^2^ of B_0.125_Er_0.125_AlN and Mg_0.125_Ti_0.125_AlN are both higher than that of Sc_0.25_Al_0.75_N, except for Be_0.125_Ce_0.125_AlN, due to the high permittivity according to Equation (1).

## 5. Conclusions

Based on the high-throughput workflow, more than 117 X_0.125_Y_0.125_Al_0.75_N compounds were examined. In addition, B_0.125_Er_0.125_Al_0.75_N, Mg_0.125_Ti_0.125_Al_0.75_N, and Be_0.125_Ce_0.125_Al_0.75_N were screened out as having a higher *e*_33_, *C*_33_, and *d*_33_ than Sc_0.25_Al_0.75_N. The *Q_r_* of the resonators made with these three systems was higher than that of Sc_0.25_AlN. The effective coupling coefficient (*K_eff_*^2^) of B_0.125_Er_0.125_AlN and Mg_0.125_Ti_0.125_AlN was also higher than that of Sc_0.25_AlN, except for Be_0.125_Ce_0.125_AlN due to the high permittivity. The *C*_33_ is affected by the electronegativity difference. There is a negative correlation between the *ΔEd* and *C*_33_. The doping elements–nitrogen bond with a small *ΔEd* leads to a larger elastic constant *C*_33_ of the doped-AlN because the strength of the bond is stronger. The *e*_33_ is affected by the *du/dε* of the doping atoms. The large *du/dε* comes from the competition between the tetrahedra coordinates [AlN4] of w-AlN and the non-tetrahedra coordinates of the doping elements with d-/f- electrons. This work provides a new way to find promising doped-AlN materials for 5G filters.

## Figures and Tables

**Table 1:** Properties of the *e*_33_, *C*_33_, *d*_33_, and band gap of the 67 dopants considered in this study.

Group	Chemical Formula	*C*_33_ (GPa)	*e*_33_ (C/m^2^)	*d*_33_ (pC/N)	Band Gap (eV)
IA(X) + VA/VB(Y)	Li_0.125_As_0.125_Al_0.75_N	294.397	1.539	5.227	1.026
	Li_0.125_Nb_0.125_Al_0.75_N	224.598	2.221	9.890	1.809
	Li_0.125_Sb_0.125_Al_0.75_N	183.151	0.155	0.849	1.234
	Li_0.125_Ta_0.125_Al_0.75_N	245.201	2.242	9.143	2.177
	Na_0.125_Ta_0.125_Al_0.75_N	177.679	1.740	9.793	1.950
	K_0.125_Nb_0.125_Al_0.75_N	258.451	0.961	3.717	1.308
	K_0.125_Ta_0.125_Al_0.75_N	213.903	0.064	0.301	1.704
	Rb_0.125_Ta_0.125_Al_0.75_N	222.589	0.704	3.165	1.095
	Rb_0.125_V_0.125_Al_0.75_N	249.936	1.037	4.151	1.011
IIA(X) + IVA/IVB(Y)	Be_0.125_C_0.125_Al_0.75_N	346.605	1.749	4.627	1.808
	Be_0.125_Ce_0.125_Al_0.75_N	271.992	2.115	7.776	1.434
	Be_0.125_Ge_0.125_Al_0.75_N	350.546	1.195	3.408	3.149
	Be_0.125_Hf_0.125_Al_0.75_N	288.143	1.985	6.888	3.504
	Be_0.125_Pb_0.125_Al_0.75_N	326.451	1.224	3.748	1.573
	Be_0.125_Si_0.125_Al_0.75_N	356.115	1.176	3.303	3.959
	Be_0.125_Sn_0.125_Al_0.75_N	328.447	1.508	4.591	2.490
	Be_0.125_Ti_0.125_Al_0.75_N	294.069	2.042	6.945	3.098
	Be_0.125_Zr_0.125_Al_0.75_N	274.419	2.042	7.440	3.471
	Mg_0.125_C_0.125_Al_0.75_N	317.855	1.641	5.164	2.604
	Mg_0.125_Ce_0.125_Al_0.75_N	247.040	1.808	7.317	1.050
	Mg_0.125_Ge_0.125_Al_0.75_N	314.839	1.492	4.740	2.355
	Mg_0.125_Hf_0.125_Al_0.75_N	245.935	2.215	9.008	3.124
	Mg_0.125_Pb_0.125_Al_0.75_N	294.874	1.544	5.238	1.025
	Mg_0.125_Si_0.125_Al_0.75_N	321.218	1.632	5.081	2.891
	Mg_0.125_Sn_0.125_Al_0.75_N	304.080	1.545	5.080	2.273
	Mg_0.125_Ti_0.125_Al_0.75_N	261.105	2.408	9.223	2.744
	Mg_0.125_Zr_0.125_Al_0.75_N	243.235	2.180	8.962	2.947
	Ca_0.125_Ce_0.125_Al_0.75_N	253.372	1.484	5.858	1.282
	Ca_0.125_Ge_0.125_Al_0.75_N	258.318	1.549	5.995	1.677
	Ca_0.125_Hf_0.125_Al_0.75_N	260.880	1.660	6.363	2.644
	Ca_0.125_Pb_0.125_Al_0.75_N	252.145	1.440	5.712	0.532
	Ca_0.125_Si_0.125_Al_0.75_N	291.283	1.595	5.477	2.523
	Ca_0.125_Sn_0.125_Al_0.75_N	256.727	1.628	6.340	1.549
	Ca_0.125_Ti_0.125_Al_0.75_N	259.020	1.841	7.107	2.370
	Ca_0.125_Zr_0.125_Al_0.75_N	219.511	1.899	8.650	2.425
	Sr_0.125_Ge_0.125_Al_0.75_N	239.683	0.161	0.672	1.509
	Sr_0.125_Hf_0.125_Al_0.75_N	182.913	0.595	3.251	1.400
	Sr_0.125_Si_0.125_Al_0.75_N	276.980	0.466	1.682	2.327
	Sr_0.125_Sn_0.125_Al_0.75_N	257.722	0.950	3.687	1.620
	Sr_0.125_Ti_0.125_Al_0.75_N	202.510	1.440	7.112	1.597
	Sr_0.125_Zr_0.125_Al_0.75_N	265.455	1.358	5.114	1.824
	Ba_0.125_C_0.125_Al_0.75_N	173.168	1.280	7.393	1.644
	Ba_0.125_Ce_0.125_Al_0.75_N	240.387	0.850	3.538	0.973
	Ba_0.125_Hf_0.125_Al_0.75_N	217.607	0.836	3.841	1.757
	Ba_0.125_Si_0.125_Al_0.75_N	278.275	0.444	1.596	1.423
	Ba_0.125_Sn_0.125_Al_0.75_N	309.324	0.520	1.682	0.382
	Ba_0.125_Ti_0.125_Al_0.75_N	227.211	1.289	5.672	1.582
	Ba_0.125_Zr_0.125_Al_0.75_N	165.556	0.745	4.497	0.929
IIIA/IIIB(X) + IIIA/IIIB(Y)	B_0.125_Er_0.125_Al_0.75_N	262.248	2.112	8.052	2.883
	B_0.125_Ga_0.125_Al_0.75_N	396.671	1.202	3.030	3.533
	B_0.125_La_0.125_Al_0.75_N	253.881	0.683	2.690	1.918
	B_0.125_Sc_0.125_Al_0.75_N	309.808	1.888	6.093	3.005
	B_0.125_Y_0.125_Al_0.75_N	284.759	2.045	7.180	2.659
	Sc_0.125_Ga_0.125_Al_0.75_N	300.226	1.543	5.141	3.532
	Sc_0.125_La_0.125_Al_0.75_N	249.583	1.440	5.769	2.098
	Sc_0.125_Y_0.125_Al_0.75_N	222.807	2.026	9.092	2.729
	Er_0.125_Ga_0.125_Al_0.75_N	293.187	1.359	4.634	2.848
	Er_0.125_La_0.125_Al_0.75_N	273.622	1.229	4.490	1.972
	Er_0.125_Sc_0.125_Al_0.75_N	225.194	1.877	8.337	2.788
	Er_0.125_Y_0.125_Al_0.75_N	231.736	1.706	7.362	2.339
	In_0.125_B_0.125_Al_0.75_N	349.798	1.342	3.837	2.462
	In_0.125_Ga_0.125_Al_0.75_N	348.935	1.260	3.611	2.844
	In_0.125_Sc_0.125_Al_0.75_N	281.114	1.624	5.778	2.898
	In_0.125_Y_0.125_Al_0.75_N	271.097	1.404	5.180	2.318
	La_0.125_Ga_0.125_Al_0.75_N	270.619	1.368	5.056	2.001
	Y_0.125_Ga_0.125_Al_0.75_N	306.706	1.418	4.623	2.886
	Y_0.125_La_0.125_Al_0.75_N	265.515	1.407	5.298	1.950
	w-AlN	359.862	1.471	4.087	4.056
	Sc_0.25_Al_0.75_N	249.592	1.869	7.488	3.287

**Table 2:** Resonant characteristics of the resonator based on doped/undoped w-AlN. *f_s_* and *f_p_* represent resonant frequency and anti-resonant frequency. *K_eff_*^2^ and *Q_r_* are calculated by COMSOL software. *k*_33_^2^ is calculated according to Equation (1).

Piezoelectric Materials	*f_s_* (GHz)	*f_p_* (GHz)	*Q_r_* (None)	*K_eff_*^2^ (None)	*k*_33_^2^ (None)
w-AlN	5.237	5.348	1603.288	0.050	0.063
B_0.125_Er_0.125_Al_0.75_N	4.696	4.945	1420.659	0.118	0.143
Be_0.125_Ce_0.125_Al_0.75_N	4.763	4.941	1438.494	0.086	0.100
Mg_0.125_Ti_0.125_Al_0.75_N	4.707	5.010	1434.593	0.140	0.177
Sc_0.25_Al_0.75_N	4.632	4.846	1407.990	0.104	0.123

## Data Availability

Not applicable.

## Supplementary Materials

The following supporting information can be downloaded at: https://www.mdpi.com/article/10.3390/ma16051778/s1, Figure S1: The two-dimensional sandwich structure of the resonator; Figure S2: The calculated and experimented *e*_33_, *C*_33_, and *d*_33_ of Sc_x_Al_1−x_N (x = 0~0.5); Figure S3: (a,b) Wave function analyses of Li_0.125_Ta_0.125_Al_0.75_N and Li_0.125_Sb_0.125_Al_0.75_N; Table S1: Physical parameters of the materials utilized in the simulation; Table S2: Dopants considered in this study and the result of *C*_11_-*C*_12_, 2*C*_13_^2^-*C*_33_(*C*_11_ + *C*_12_), *C*_66_. Reference [48] are cited in the supplementary materials.
