# Supplementary material for: Doping Engineering for Optimizing Piezoelectric and Elastic Performance of AlN
Source: Materials (Basel). 2023 Feb 21;16(5):1778. doi: 10.3390/ma16051778 (PMC10004460; doi:10.3390/ma16051778)
Supplement: Supplementary file 1 [file materials-16-01778-s001.zip › materials-2078526-supplementary.pdf]

Doping Engineering for Optimizing Piezoelectric and Elastic Performance of AlN

Xi Yu [1,†], Lei Zhu [2,†], Xin Li [3], Jia Zhao [2], Tingjun Wu [2], Wenjie Yu [2,3] and Weimin Li [1,2,3,*]

[1] School of Microelectronics, Shanghai University, Shanghai 201899, China; yuxi666@shu.edu.cn
[2] State Key Laboratory of Functional Materials for Informatics, Shanghai Institute of Microsystem and Information Technology, Chinese Academy of Sciences, Shanghai 200050, China; leizhu@mail.sim.ac.cn (L.Z.); zhaojia@mail.sim.ac.cn (J.Z.); tjwu@mail.sim.ac.cn (T.W.); casan@mail.sim.ac.cn (W.Y.)
[3] Shanghai Institute of IC Materials Co., Ltd., Shanghai 201899, China; xinli@sicm.com.cn
* Correspondence: weimin.li@mail.sim.ac.cn
† These authors contributed equally to this work.

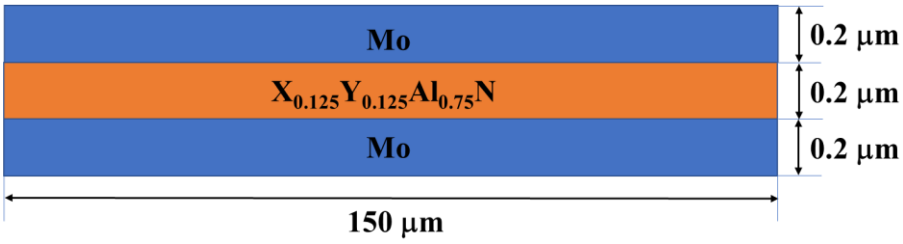

Figure S1 The two-dimensional sandwich structure of the resonator.

Table S1. Physical parameters of materials utilized in simulation

Piezoelectric materials	Density(kg/m^3)	e_{33}(C/m^2)	C_{33}(Pa)	Relative permittivity
$Be_{0.125}Ce_{0.125}AlN$	3843.200	2.115	2.720E+11	16.369
$B_{0.125}Er_{0.125}AlN$	4209.300	2.112	2.622E+11	11.847
$Mg_{0.125}Ti_{0.125}AlN$	3225.100	2.408	2.611E+11	12.510
$Sc_{0.25}AlN$	3219.900	1.869	2.495E+11	11.360
AlN	3205.100	1.471	3.598E+11	9.585

The mechanically criterion was tested by Born-Huang criteria of hexagonal structure[1]: $C_{11}>C_{12}$, $2C_{13}^2<C_{33}(C_{11}+C_{12})$, $C_{44}>0$, $C_{66}>0$. It is clear that all of the models we considered are mechanically stable, and detailed results are listed in Table S2.

Table S2. Dopants considered in this study and the results of C_{11}-C_{12}, $2C_{13}^2$- $C_{33}(C_{11}+C_{12})$, C_{66}.

Group	Chemical formula	C_{11}-C_{12} (GPa)	$2C_{13}^2$- $C_{33}(C_{11}+C_{12})$ (GPa²)	C_{66} (GPa)	*If stable*
IA(X) + VA/VB(Y)	$Li_{0.125}As_{0.125}Al_{0.75}N$	217.422	-112853.599	94.981	√
	$Li_{0.125}Nb_{0.125}Al_{0.75}N$	179.625	-71698.154	88.215	√
	$Li_{0.125}Sb_{0.125}Al_{0.75}N$	192.476	-61642.424	91.658	√
	$Li_{0.125}Ta_{0.125}Al_{0.75}N$	180.820	-84021.593	97.728	√
	$Na_{0.125}Ta_{0.125}Al_{0.75}N$	154.334	-44263.793	83.001	√
	$K_{0.125}Nb_{0.125}Al_{0.75}N$	124.384	-69973.078	66.850	√
	$K_{0.125}Ta_{0.125}Al_{0.75}N$	137.523	-52000.058	69.423	√
	$Rb_{0.125}Ta_{0.125}Al_{0.75}N$	127.566	-47646.108	72.332	√
	$Rb_{0.125}V_{0.125}Al_{0.75}N$	139.538	-70238.711	87.125	√
IIA(X) + IVA/IVB(Y)	$Be_{0.125}C_{0.125}Al_{0.75}N$	159.475	-116695.581	91.849	√
	$Be_{0.125}Ce_{0.125}Al_{0.75}N$	121.725	-61017.664	73.101	√
	$Be_{0.125}Ge_{0.125}Al_{0.75}N$	256.600	-157231.279	111.159	√
	$Be_{0.125}Hf_{0.125}Al_{0.75}N$	201.073	-107150.023	103.099	√
	$Be_{0.125}Pb_{0.125}Al_{0.75}N$	214.989	-126974.964	93.005	√
	$Be_{0.125}Si_{0.125}Al_{0.75}N$	250.586	-160800.695	111.201	√
	$Be_{0.125}Sn_{0.125}Al_{0.75}N$	231.171	-136327.764	101.125	√
	$Be_{0.125}Ti_{0.125}Al_{0.75}N$	216.891	-115798.731	105.903	√
	$Be_{0.125}Zr_{0.125}Al_{0.75}N$	196.193	-97386.926	98.910	√
	$Mg_{0.125}C_{0.125}Al_{0.75}N$	196.618	-116767.714	105.300	√
	$Mg_{0.125}Ce_{0.125}Al_{0.75}N$	126.932	-65843.005	71.791	√
	$Mg_{0.125}Ge_{0.125}Al_{0.75}N$	226.452	-127379.233	102.066	√
	$Mg_{0.125}Hf_{0.125}Al_{0.75}N$	180.075	-80807.221	95.735	√
	$Mg_{0.125}Pb_{0.125}Al_{0.75}N$	195.192	-106048.038	86.251	√
	$Mg_{0.125}Si_{0.125}Al_{0.75}N$	231.206	-133641.743	108.042	√
	$Mg_{0.125}Sn_{0.125}Al_{0.75}N$	210.925	-118339.501	95.462	√
	$Mg_{0.125}Ti_{0.125}Al_{0.75}N$	199.802	-95314.837	99.020	√
	$Mg_{0.125}Zr_{0.125}Al_{0.75}N$	187.514	-81809.303	92.272	√
	$Ca_{0.125}Ce_{0.125}Al_{0.75}N$	104.665	-62731.181	59.206	√
	$Ca_{0.125}Ge_{0.125}Al_{0.75}N$	179.259	-81768.671	80.804	√
	$Ca_{0.125}Hf_{0.125}Al_{0.75}N$	157.504	-77299.133	79.034	√
	$Ca_{0.125}Pb_{0.125}Al_{0.75}N$	152.658	-73682.705	71.576	√
	$Ca_{0.125}Si_{0.125}Al_{0.75}N$	193.790	-98906.248	87.347	√
	$Ca_{0.125}Sn_{0.125}Al_{0.75}N$	166.095	-80000.920	80.412	√
	$Ca_{0.125}Ti_{0.125}Al_{0.75}N$	164.901	-88012.165	87.630	√
	$Ca_{0.125}Zr_{0.125}Al_{0.75}N$	137.779	-53745.238	75.533	√
	$Sr_{0.125}Ge_{0.125}Al_{0.75}N$	151.658	-70739.473	65.425	√
	$Sr_{0.125}Hf_{0.125}Al_{0.75}N$	121.972	-38055.532	62.935	√
	$Sr_{0.125}Si_{0.125}Al_{0.75}N$	178.822	-88722.725	80.937	√

	$Sr_{0.125}Sn_{0.125}Al_{0.75}N$	149.516	-72220.820	80.124	√
	$Sr_{0.125}Ti_{0.125}Al_{0.75}N$	139.152	-49786.625	72.739	√
	$Sr_{0.125}Zr_{0.125}Al_{0.75}N$	137.085	-72930.183	69.771	√
	$Ba_{0.125}C_{0.125}Al_{0.75}N$	162.708	-36438.610	84.831	√
	$Ba_{0.125}Ce_{0.125}Al_{0.75}N$	81.059	-56504.127	58.995	√
	$Ba_{0.125}Hf_{0.125}Al_{0.75}N$	125.097	-51771.349	75.076	√
	$Ba_{0.125}Si_{0.125}Al_{0.75}N$	149.030	-78398.380	91.476	√
	$Ba_{0.125}Sn_{0.125}Al_{0.75}N$	132.316	-97377.052	64.345	√
	$Ba_{0.125}Ti_{0.125}Al_{0.75}N$	132.633	-52026.077	82.655	√
	$Ba_{0.125}Zr_{0.125}Al_{0.75}N$	134.349	-41246.734	42.632	√
IIIA/IIIB(X) + IIIA/IIIB(Y)	$B_{0.125}Er_{0.125}Al_{0.75}N$	135.400	-79236.333	80.369	√
	$B_{0.125}Ga_{0.125}Al_{0.75}N$	262.601	-187115.965	115.669	√
	$B_{0.125}La_{0.125}Al_{0.75}N$	152.927	-83277.217	97.025	√
	$B_{0.125}Sc_{0.125}Al_{0.75}N$	174.276	-110146.273	106.033	√
	$B_{0.125}Y_{0.125}Al_{0.75}N$	172.711	-89167.692	90.901	√
	$Sc_{0.125}Ga_{0.125}Al_{0.75}N$	203.343	-112536.751	93.887	√
	$Sc_{0.125}La_{0.125}Al_{0.75}N$	116.371	-66158.658	70.979	√
	$Sc_{0.125}Y_{0.125}Al_{0.75}N$	136.847	-55648.047	79.537	√
	$Er_{0.125}Ga_{0.125}Al_{0.75}N$	182.802	-98097.040	86.268	√
	$Er_{0.125}La_{0.125}Al_{0.75}N$	116.700	-75347.453	66.326	√
	$Er_{0.125}Sc_{0.125}Al_{0.75}N$	166.965	-64324.952	81.906	√
	$Er_{0.125}Y_{0.125}Al_{0.75}N$	122.063	-53059.318	72.057	√
	$In_{0.125}B_{0.125}Al_{0.75}N$	200.190	-133514.877	98.772	√
	$In_{0.125}Ga_{0.125}Al_{0.75}N$	218.181	-144321.591	97.042	√
	$In_{0.125}Sc_{0.125}Al_{0.75}N$	178.166	-98377.421	88.966	√
	$In_{0.125}Y_{0.125}Al_{0.75}N$	160.289	-84553.634	77.894	√
	$La_{0.125}Ga_{0.125}Al_{0.75}N$	155.340	-86234.389	71.685	√
	$Y_{0.125}Ga_{0.125}Al_{0.75}N$	174.196	-110884.945	87.588	√
	$Y_{0.125}La_{0.125}Al_{0.75}N$	110.666	-72513.420	66.562	√

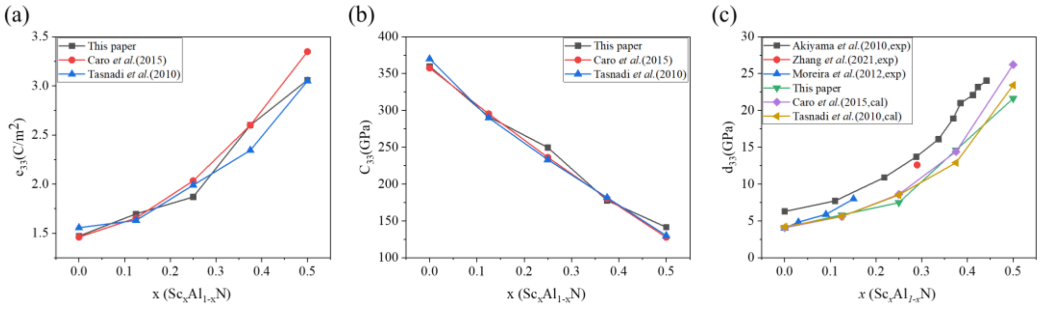

Figure S2. The calculated and experimented (a)e_{33}, (b)C_{33} and (c)d_{33} of $Sc_xAl_{1-x}N$ ($x = 0{\sim}0.5$). Our calculated e_{33} and C_{33} of $Sc_xAl_{1-x}N$ ($x = 0{\sim}0.5$) are consistent with the reported values of Caro et al. [2], Tasnadi et al. [3], Akiyama et al.[4], Zhang et al.[5] and Moreira et al.[6].

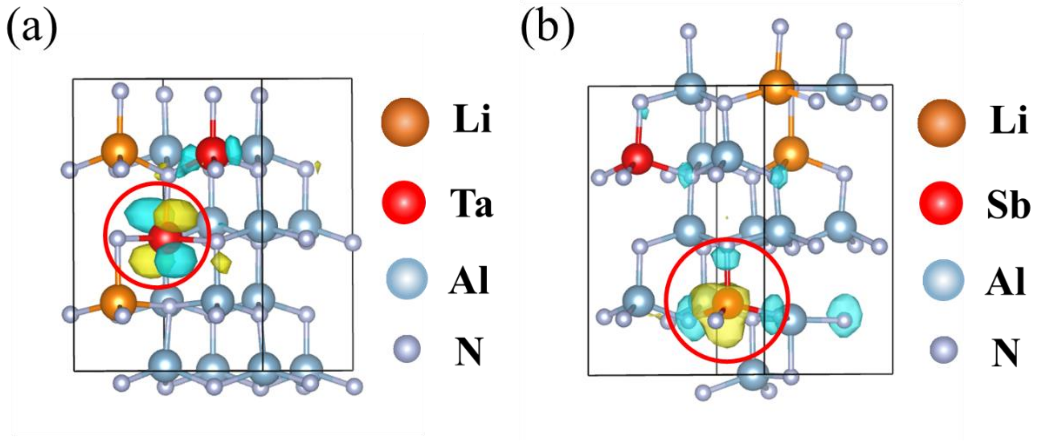

Figure S3. (a-b) Wave function analyses of $Li_{0.125}Ta_{0.125}Al_{0.75}N$ and $Li_{0.125}Sb_{0.125}Al_{0.75}N$. Blue represents bonding orbitals, yellow represents anti-bonding orbitals.

The discussion viscosity coefficient

For acoustical materials, after introducing mechanical losses and a positive idle steady-state response, the constitutive equation [7] can become

$$\begin{gathered} \boldsymbol{T} = \boldsymbol{c}:\boldsymbol{S} + \mathrm{j}\omega\boldsymbol{\eta} \rightarrow \boldsymbol{c}':\boldsymbol{S} \\ \boldsymbol{c}' = \boldsymbol{c} + \mathrm{j}\omega\boldsymbol{\eta} \end{gathered} \tag{S1}$$

The mechanical loss is represented by the viscosity coefficient $\boldsymbol{\eta}$, which is the same fourth order tensor as the elastic stiffness constant $\boldsymbol{c}$. At the same time, $\boldsymbol{c}'$ can be expressed as

$$\boldsymbol{c}' = (\mathbf{1} + \mathrm{j}\boldsymbol{\eta_s})\boldsymbol{c} \tag{S2}$$

where $\boldsymbol{\eta}_s$ is Isotropic structural loss factor. With equations 2 and 3 it can be deduced that

$$\boldsymbol{\eta} = \frac{\eta_s \boldsymbol{c}}{\omega} \tag{S3}$$

The mechanism of C_{33}

The hardness of crystal with n types of bonds can be expressed as [8] [9] [10],

$$H_k(GPa) = 423.8n\left[\prod_{a,b=1}^{n} \frac{N_{ab}}{V} X_{ab} e^{-2.7f_{i(ab)}}\right]^{\frac{1}{n}} - 3.4 \tag{S4}$$

$$X_{ab} = \sqrt{\frac{\chi_a}{CN_a}\frac{\chi_b}{CN_b}} \tag{S5}$$

$$f_i = \frac{\frac{1}{2}|\chi_a - \chi_b|}{2\sqrt{\chi_a \chi_b}} \tag{S6}$$

where f_i (i=1,2,3,⋯⋯n), N_{ab}/V, X_j, and CN_j (j=a,b) represent ionicity indicator, the density of covalent bond a-b, electronegativity (EN), and coordination numbers of atom a or b. The covalent bond is composed of ($1/CN_a$) a atom and ($1/CN_b$) b atom. The hardness of crystal is positively related to X_{ab} and bond density N_{ab}/V and negatively related to the ionicity indicator f_i.

For typical multi-bond crystals $X_{0.125}Y_{0.125}Al_{0.75}N$, the hardness can be expressed as a geometrical sum of all binary bonds, X-N bond, Y-N bond and the Al-N bond. Due to the EN of all doping atoms we choose in this paper are smaller than N, thus the EN of X and Y larger,

the electronegativity difference smaller, and the C_{33} higher. For doping elements only has s- and p- electrons, they tend to format tetrahedral coordination as Al due to the sp3 hybridization. Only transition elements with d- or f- electrons tend to format non-tetrahedral (such as octahedral for Ti_3N_4). However, due to X and Y atoms are doped into Al sites, there are only four N atoms around X and Y for bonding. Thus the influence of CN_j on C_{33} could be neglected. However, octahedral coordination atoms doped in the Al sites will produce lattice distortion. Moreover, the atom radius difference between doping atoms and substituted Al atoms also may produce lattice distortion. This effect can be consolidated by slight changes in the bond density. In general, the hardness of crystal is mainly affected by the ionicity indicator f_i and slightly affected by the bond density N_{ab}/V induced by the small lattice distortion.

References

1. Born, M.; Huang, K.; Lax, M. Dynamical Theory of Crystal Lattices. *American Journal of Physics* **1955**, *23*, 474–474, doi:10.1119/1.1934059.
2. Caro, M.A.; Zhang, S.; Riekkinen, T.; Ylilammi, M.; Moram, M.A.; Lopez-Acevedo, O.; Molarius, J.; Laurila, T. Piezoelectric Coefficients and Spontaneous Polarization of ScAlN. *J. Phys.: Condens. Matter* **2015**, *27*, 245901, doi:10.1088/0953-8984/27/24/245901.
3. Tasnádi, F.; Alling, B.; Höglund, C.; Wingqvist, G.; Birch, J.; Hultman, L.; Abrikosov, I.A. Origin of the Anomalous Piezoelectric Response in Wurtzite ScxAl1-XN Alloys. *Phys. Rev. Lett.* **2010**, *104*, 137601, doi:10.1103/PhysRevLett.104.137601.
4. Akiyama, M.; Kano, K.; Teshigahara, A. Influence of Growth Temperature and Scandium Concentration on Piezoelectric Response of Scandium Aluminum Nitride Alloy Thin Films. *Appl. Phys. Lett.* **2009**, *95*, 162107, doi:10.1063/1.3251072.
5. Zhang, Q.; Chen, M.; Liu, H.; Zhao, X.; Qin, X.; Wang, F.; Tang, Y.; Yeoh, K.H.; Chew, K.-H.; Sun, X. Deposition, Characterization, and Modeling of Scandium-Doped Aluminum Nitride Thin Film for Piezoelectric Devices. *Materials* **2021**, *14*, 6437, doi:10.3390/ma14216437.
6. Moreira, M.A.; Bjurström, J.; Yantchev, V.; Katardjiev, I. Synthesis and Characterization of Highly C-Textured Al(1-x)Sc(x)N Thin Films in View of Telecom Applications. *IOP Conf. Ser.: Mater. Sci. Eng.* **2012**, *41*, 012014, doi:10.1088/1757-899X/41/1/012014.
7. Auld, B.A. *Acoustic Fields and Waves in Solids*; Jone Wiley & Sons, 1973;
8. Sanderson, R.T. An Interpretation of Bond Lengths and a Classification of Bonds. *Science* **1951**, *114*, 670–672, doi:10.1126/science.114.2973.670.
9. Li, K.; Wang, X.; Zhang, F.; Xue, D. Electronegativity Identification of Novel Superhard Materials. *Phys. Rev. Lett.* **2008**, *100*, 235504, doi:10.1103/PhysRevLett.100.235504.
10. Gao, F.; He, J.; Wu, E.; Liu, S.; Yu, D.; Li, D.; Zhang, S.; Tian, Y. Hardness of Covalent Crystals. *Phys. Rev. Lett.* **2003**, *91*, 015502, doi:10.1103/PhysRevLett.91.015502.
